# Haemorrhagic Colitis Caused by Dasatinib

**DOI:** 10.1155/2012/417106

**Published:** 2012-12-22

**Authors:** Nishant Patodi, Nidhi Sagar, Zbigniew Rudzki, Gerald Langman, Naveen Sharma

**Affiliations:** ^1^Department of Gastroenterology, Heart of England Foundation Trust, Birmingham B9 5SS, UK; ^2^Department of Histopathology, Heart of England Foundation Trust, Birmingham B9 5SS, UK

## Abstract

Gastrointestinal bleeding appears to be a common adverse event associated with dasatinib therapy. Here we present a case of a 59-year-old man with chronic myeloid leukaemia (CML) developing the rarest complication of haemorrhagic colitis with dasatinib therapy which resolved rapidly after treatment withdrawal.

## 1. Introduction

Dasatinib is a widely used therapy for chronic myeloid leukaemia especially when resistance has occurred to first line therapies such as Imatinib [1]. Although gastrointestinal bleeding has been widely reported with dasatinib therapy for CML, haemorrhagic colitis appears to be a much rarer event [2]. Here we describe the previous reports of haemorrhagic colitis associated with dasatinib therapy and describe some of the mechanisms which may be important.

## 2. Case Report

**Figure 1:**
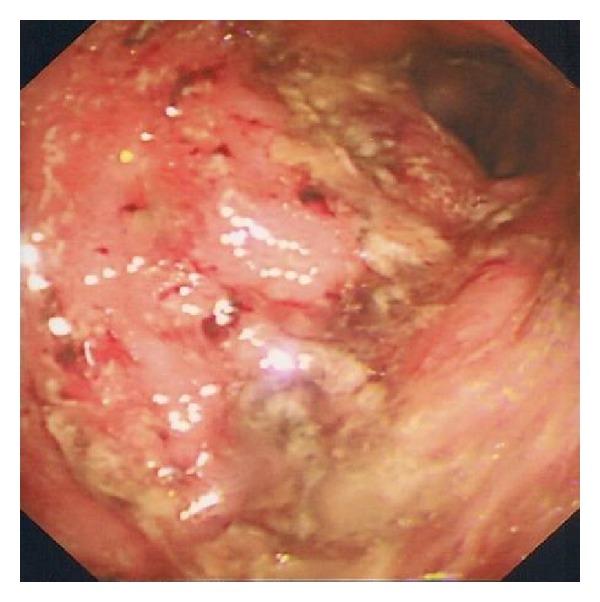
Endoscopic image of the sigmoid colon demonstrating deep ulceration with mucopurulent exudate.

A 59-year-old man with chronic-phase chronic myeloid leukaemia (CML), first diagnosed in 2004, was initially treated with Imatinib 400–800 mg once a day. He developed cytogenic resistance to Imatinib and was switched to dasatinib 70 mg twice a day, achieving a good haematological response. Mutation analysis revealed an E450Q mutation in the ABL kinase domain of the BCR ABL fusion gene which has been shown to be associated with Imatinib resistance [3]. Three years after initiation of treatment with dasatinib the patient was admitted to hospital with a two-month history of diarrhoea (bowel frequency 8 times/24 hours), rectal bleeding, and weight loss. He was not on any other medications prior to admission. On examination, the patient appeared dehydrated and had a sinus tachycardia. Initial investigations revealed a haemoglobin of 9 g/dL, platelet count of 110 × 10^9^/L, the total and differential white cell count and prothrombin time were within normal limits. Serum albumin was significantly reduced at 24 g/L and serum CRP was significantly raised at 99 mg/L. Stool cultures were negative for Clostridium difficile and other pathogenic enteric bacteria. Endoscopic examination of the lower gastrointestinal tract revealed a granular and congested mucosa in the rectum along with large ulcers between the descending colon and splenic flexure (see Figure 1). The possibility of ulcerative colitis was raised and he was commenced on high-dose intravenous hydrocortisone and mesalasine. He responded well clinically to this treatment combination and was discharged home on a tapering course of prednisolone. 

**Figure 2:**
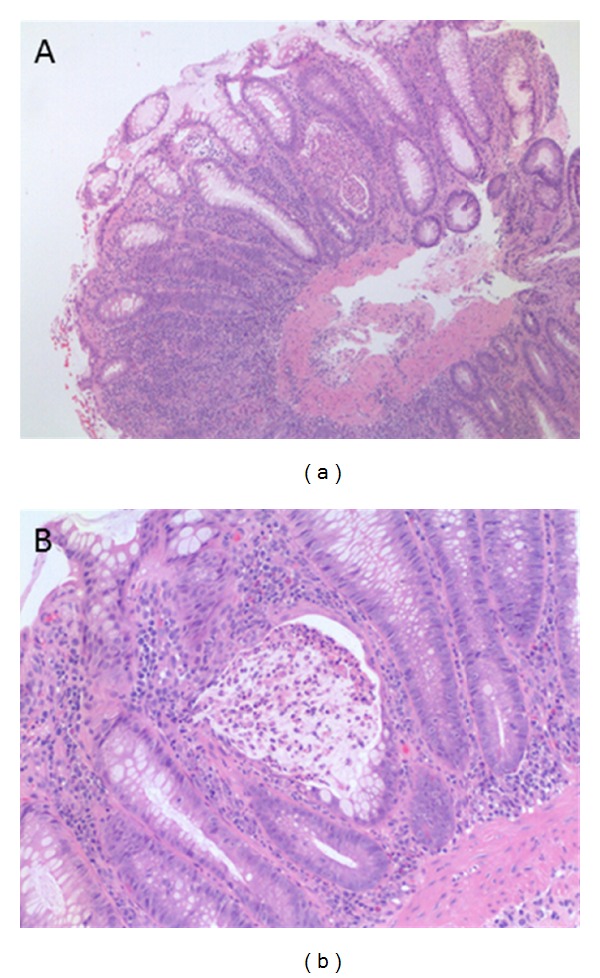
(a) A diffuse inflammatory cell infiltrate is present in the lamina propria with cryptitis (H&E; ×40). (b) A crypt is distended by mucus and neutrophils while the lining colonocytes are focally attenuated (H&E; ×100).

Histology revealed an acute colitis. The crypt architecture was well preserved and this argued against a diagnosis of inflammatory bowel disease. There was no leukaemic involvement and given the clinical presentation a drug aetiology was raised (see Figures 2(a) and 2(b)).

**Figure 3:**
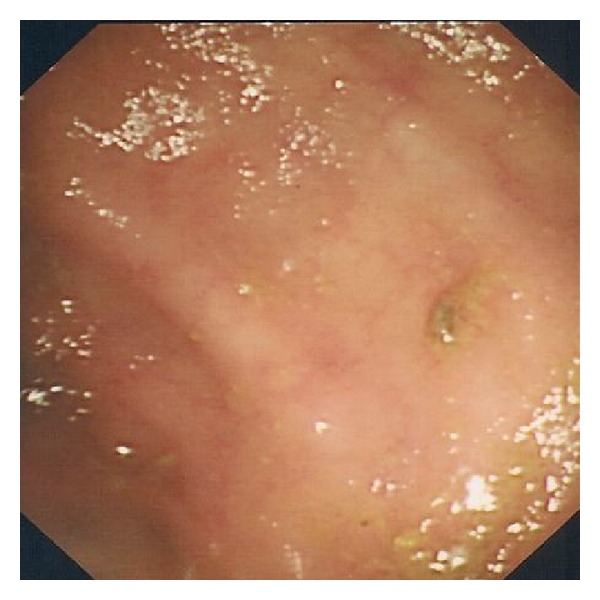
Resolution of colonic ulceration following discontinuation of dasatinib.

The patient was readmitted two months after discharge with worsening diarrhoea, rectal bleeding, anorexia, and weight loss. He was restarted on Prednisolone 30 mg daily, dasatinib was stopped, and nilotinib was started as an alternative treatment for CML. Seven days after withdrawal of dasatinib he was asymptomatic—opening his bowels once per day with no further rectal bleeding. Endoscopic examination was repeated two weeks later and no ulceration was noted (see Figure 3). Histology revealed inflammation but to a lesser degree compared to the previous examination. He was reviewed in clinic a month later and had made a complete recovery. He remained well off steroids some six months later.

## 3. Discussion

Dasatinib is an oral tyrosine kinase inhibitor which inhibits several kinases including BCR-ABL kinase [4]. It was initially approved for the treatment of all phases of CML (chronic, accelerated, or blast phase) for patients who demonstrated resistance or intolerance to prior therapy with Imatinib [1]. It has recently gained a licensing extension for use as first-line treatment of adults with newly diagnosed Philadelphia chromosome-positive (Ph+) CML in the chronic phase [5]. 

Dasatinib therapy is generally well tolerated but side effects such as myelosuppression, peripheral oedema, skin rashes, and gastrointestinal symptoms have been reported [6]. Commonly reported gastrointestinal side effects include diarrhoea, nausea, vomiting, anorexia, and gastrointestinal bleeding. Gastrointestinal bleeding is thought to account for 14–26% of gastrointestinal adverse events due to dasatinib [2].

The variables thought to increase the risk of bleeding whilst on dasatinib therapy include thrombocytopenia (platelets <30 × 10^9^/L), duration of CML, and advanced disease (bleeding noted more commonly in the accelerated and blast phase of CML as opposed to the chronic phase of CML) [2]. Our patient was in the accelerated phase of his disease and had been diagnosed with CML seven years before onset of his symptoms. He was also noted to be thrombocytopenic (platelet count 53–110) during his hospital admission.

The causes of bleeding secondary to dasatinib are likely to be multifactorial and the underlying pathophysiology remains poorly understood. Dasatinib has been shown to significantly reduce platelet aggregation in CML patients when compared to the use of other tyrosine kinase inhibitors [7]. A further study demonstrated that *in vitro* platelet activation by collagen was reduced and tail bleeding in mice was increased following dasatinib exposure [8]. This study also demonstrated that withdrawal of dasatinib led to a rapid reversal of these effects. In addition, dasatinib is a potent inhibitor of platelet derived growth factor receptor (PDGFR) kinase [9]. PDGFR null mice have defective *in utero* angiogenesis and capillary wall development leading to microaneurysm formation and haemorrhage [10]. 

Mustjoki et al. described an association between lymphocytosis secondary to significant expansion of clonal large granular lymphocytes (LGL) and patients receiving dasatinib [11]. Dasatinib-induced complications such as colitis and pleural effusions have developed in a marked proportion of patients found to have LGL expansion and therefore clonal LGL expansion may play a potential pathogenic role in bleeding induced by dasatinib [11].

A more recent report looking at bleeding diathesis in CML patients receiving dasatinib therapy noted that 81% of all bleeding episodes were confined to the gastrointestinal tract [12]. Gastrointestinal bleeding is consistent with the oral route of dasatinib which is eliminated in the faeces. Therefore, the lower gastrointestinal tract may be particularly vulnerable after being exposed to dasatinib during its elimination.

Here we describe a case of acute haemorrhagic colitis secondary to dasatinib therapy. Current literature describing this association is scarce with only four cases being previously reported. There first case is a paediatric patient with Philadelphia acute lymphoblastic leukaemia who similarly developed haemorrhagic colitis during dasatinib therapy which responded to steroids and discontinuation of the drug [13]. In this case, however, the patient was not thrombocytopenic. The other two cases are adult patients with blastic phase CML who developed haemorrhagic colitis with dasatinib [14, 15]. Similar to the current case, dasatinib had been commenced some time before the development of haemorrhagic colitis and discontinuation of the therapy led to rapid resolution of symptoms and colonic ulceration. More recently a case of haemorrhagic colitis was reported in a 26-year-old woman with blast crisis CML when Imatinib was switched to dasatinib [16]. After achieving remission she developed bloody diarrhoea. Although she was found to have evidence of cytomegalovirus (CMV) enterocolitis, this failed to respond to treatment with ganciclovir, despite eradication of the virus. Only after discontinuation of dasatinib did her colitis resolve.

In summary, although gastrointestinal bleeding is a recognised complication of dasatinib therapy in CML, haemorrhagic colitis is much less common but responds well to discontinuation of therapy. Physicians are advised to be astute to this particular presentation especially in patients with thrombocytopenia and advanced disease. Further studies are needed to understand the exact pathophysiology of this complication.
